# Endoscopic recanalization of a complete choledochojejunal anastomotic stricture using the “piercing technique” via an endoscopic ultrasound-guided hepaticogastrostomy approach

**DOI:** 10.1055/a-2760-9189

**Published:** 2025-12-19

**Authors:** Joshua Josef Torres, Susumu Hijioka, Yoshikuni Nagashio, Shota Harai, Daiki Yamashige, Yasuhiro Komori, Takuji Okusaka

**Affiliations:** 1 68380 Department of Hepatobiliary and Pancreatic Oncology, National Cancer Center Hospital, Tokyo, Japan; 2 Department of Internal Medicine, Silliman University Medical Center Foundation, Inc., Dumaguete City, Philippines

A 52-year-old woman, 4 months post-pancreaticoduodenectomy for pancreatic cancer, was admitted for jaundice secondary to a choledochojejunal anastomotic stricture (CJS; Fig. 1 **a, b** ). An initial attempt at enteroscopy-assisted ERCP using a colonoscope (CF-260AI, Olympus, Tokyo, Japan) led to perforation of the afferent limb, requiring emergency surgery. Following recovery, we performed EUS-guided hepaticogastrostomy (EUS-HGS). Attempts to pass through the stricture using the soft tip of a 0.025-inch guidewire (Fielder-25, Asahi Intecc, Aichi, Japan) failed because of complete stenosis. We therefore attempted to recanalize the stricture with the guidewire’s stiff end – the “piercing technique” ( Video 1 ).

**Fig. 1:**
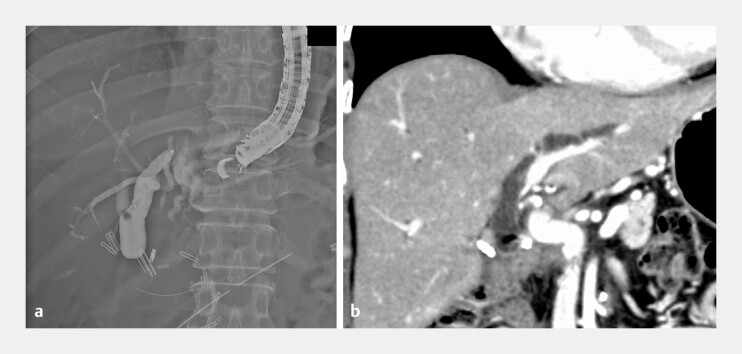
Demonstration of the choledochojejunal anastomotic stricture (CJS). **a** A cholangiogram taken via EUS-HGS, demonstrating the absence of contrast passage into the jejunum, signifying complete CJS obstruction. **b** Coronal CT scan images of the stricture reviewed in preparation for piercing, showing no intervening vessels across the stricture. CT, computed tomography; EUS-HGS, EUS-guided hepaticogastrostomy.

Endoscopic recanalization of a complete choledochojejunal anastomotic stricture using the “piercing technique” via an endoscopic ultrasound-guided hepaticogastrostomy approach.Video 1

After confirming the absence of intervening vessels across the anastomosis on computed tomography ( Fig. 1 **b** ), we advanced a guiding sheath (Endosheather; Piolax, Kanagawa, Japan) to the anastomosis, inserted the guidewire’s stiff end, aligned its axis with the CJS, and then attempted to pierce it, creating a partial tract ( Fig. 2 **a** ). Using a second guidewire, we formed a loop which was insinuated within the tract ( Fig. 2 **b** ). Continued piercing eventually achieved jejunal access ( Fig. 2 **c** ), confirmed through contrast injection ( Fig. 3 ). We dilated the tract using a 7 Fr drill dilator (Tornus-ES, Asahi Intecc, Aichi, Japan; Fig. 4 **a** ) and then a 6mm balloon dilation catheter (REN, Kaneka, Osaka, Japan; Fig. 4 **b** ). We subsequently deployed two 8 mm fully covered multi-hole SEMS (HANAROSTENT Multi-Hole Benefit, M.I.Tech, Gyeonggi-do, South Korea) side-by-side across the stricture. Finally, we deployed a 7 Fr plastic stent (Type-IT, Gadelius Medical, Tokyo, Japan) across the HGS ( Fig. 5 ). Post-procedure, there were no adverse events and her jaundice resolved.

**Fig. 2:**
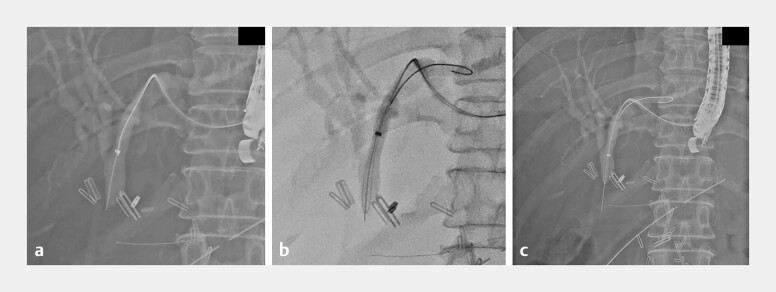
Sequential fluoroscopic images demonstrating the steps of the piercing technique on the CJS. **a** Aligning the axis of a 0.025-inch guidewire’s stiff end within a guiding sheath with the anastomotic stricture in preparation for piercing. **b** Piercing the partial tract with the stiff wire while a second looped guidewire was insinuated within the tract. **c** An image taken after successfully piercing through the CJS. CJS, choledochojejunal anastomotic stricture.

**Fig. 3:**
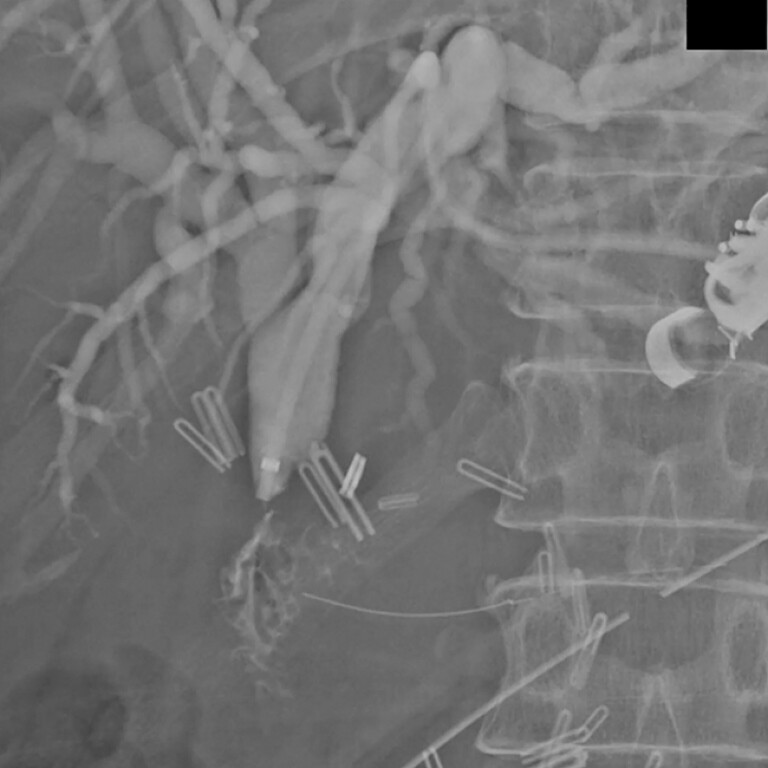
Demonstration of contrast passage through the new tract and into the jejunum to confirm intestinal access.

**Fig. 4:**
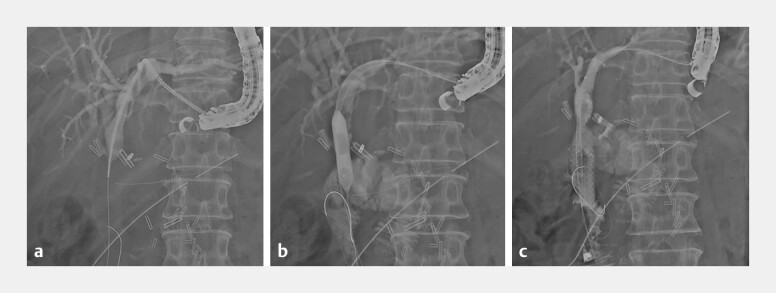
Progressive dilation of the CJS through the newly created tract. **a** Initial dilation using a 7 Fr endoscopic drill dilator. **b** Additional dilation using a 6mm biliary balloon dilation catheter. **c** Side-by-side deployment of two 8mm fully covered SEMS across the stricture. CJS, choledochojejunal anastomotic stricture.

**Fig. 5:**
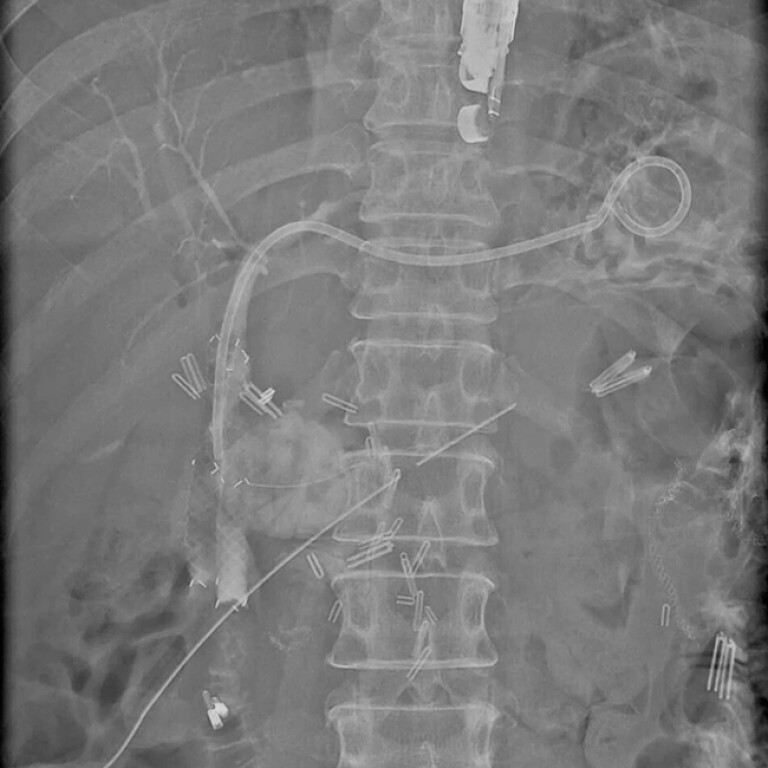
A final cholangiogram demonstrating the two fully covered SEMS across the stricture and a 7 Fr plastic stent through the HGS. HGS, hepaticogastrostomy.

The piercing technique has been reported for percutaneous and cholangioscopic recanalization of biliary strictures 1 2 3 , and for mucosal hyperplasia within SEMS after EUS-HGS 4 . To our knowledge, this is the first case of bilioenteric anastomotic stricture recanalization using the piercing technique via an HGS approach, thereby introducing a valuable alternative method in the endoscopic treatment of such strictures.

Endoscopy_UCTN_Code_TTT_1AS_2AD

## Notes

Endoscopy E-Videos https://eref.thieme.de/e-videos
*E-Videos* is an open access online section of the journal *Endoscopy* , reporting on interesting cases and new techniques in gastroenterological endoscopy. All papers include a high-quality video and are published with a Creative Commons CC-BY license. Endoscopy E-Videos qualify for HINARI discounts and waivers and eligibility is automatically checked during the submission process. We grant 100% waivers to articles whose corresponding authors are based in Group A countries and 50% waivers to those who are based in Group B countries as classified by Research4Life (see: https://www.research4life.org/access/eligibility/ ). This section has its own submission website at https://mc.manuscriptcentral.com/e-videos .
